# Pulpal and periapical tissue response after direct pulp capping with endosequence root repair material and low-level laser application

**DOI:** 10.1186/s12903-022-02099-0

**Published:** 2022-03-04

**Authors:** Loai Alsofi, Wafaa Khalil, Nada O. Binmadi, Mey A. Al-Habib, Hanan Alharbi

**Affiliations:** 1 Department of Endodontics, Faculty of Dentistry, King Abdulaziz University, P.O.Box 80209, Jeddah, 21589 Saudi Arabia; 2 Department of Oral Diagnostic Sciences, Faculty of Dentistry, King Abdulaziz University, P.O.Box 80209, Jeddah, 21589 Saudi Arabia; 3 Division of Endodontics, Department of Conservative Sciences, College of Dentistry, Qassim University, Qassim, Saudi Arabia

**Keywords:** Bioceramics, Predentin thickness, Endosequence root repair material, Laser, Direct pulp capping

## Abstract

**Background:**

The study aims to investigate the pulp and periapical reaction and healing after capping with EndoSequence Root Repair Material (ERRM) combined with low-level laser application.

**Methods:**

In 6 rabbits, pulps were exposed via class V, half of the samples received a low-level diode laser at 980 nm. Thereafter, cavities were capped with regular-set ERRM. The specimens were processed for histomorphological examination after 2 weeks and two months.

**Results:**

After 2 weeks, images show mild inflammation and organized odontoblasts in lased group. The non-lased group shows more severe inflammation. The predentin thickness was thicker in the lased group with statistical significance (*p* < 0.05). After 2 months, inflammatory cells were sparse in both lased and non-lased groups. In the periapical area, group one showed dilated blood vessels and thick fibrous connective tissues. In group two, there were more numerous maturations of PDL fibers with scattered inflammatory cells and congested blood vessel.

**Conclusions:**

Using low-level laser therapy in combination with ERRM for pulp capping shortens the inflammatory phase and enhances healing.

## Background

Direct pulp capping is a treatment modality performed when the dental pulp is exposed to preserve its vitality. The pulp capping material plays a principal role in the treatment’s success. Different animal model studies analyzed the reaction of pulpal tissues when they come in direct contact with capping materials [1].

The ideal material should allow healing of the pulpal exposure with minimal inflammatory response towards the material and successful formation of a hard tissue barrier [2]. The healing of pulpal tissues depends on the material’s biocompatibility and bioactivity [3]. The biocompatibility of the material is represented by the intensity and extent of pulpal inflammation while the bioactivity is represented by the category of hard tissue barrier formation at the interface between the material and pulpal tissue [4].

Different materials have been used as pulp capping agents, each having its advantages and drawbacks. Mineral Trioxide Aggregate [5], Bioceramics [6, 7], Biodentine [8] and, other calcium silicates [9, 10] were all reported capable of healing pulpal exposure and forming a hard dentin barrier [4, 11].

In recent years, bioceramics have been developed to overcome the drawbacks of MTA. EndoSequence root repair material (ERRM) is a bioceramic material recommended for perforation repair, pulp capping, and periapical surgery. It is aluminum-free, with a high pH, radiopaque, and has superior handling properties. It also has been shown to be similar to MTA in biocompatibility, bioactivity, proliferation of dental pulp cells, and dentinal bridge formation [3, 11, 12].

The use of lasers for direct pulp capping shows great potential with respect to clinical and basic sciences because of their adaptability and wide applicability [13].

Photobiostimulation by using diode low-level laser therapy (LLLTT) has the advantage of accelerating the healing of exposed pulp tissue capped by Ca(OH)_2_ with progressive fibroblasts proliferation and formation of tubular dentin [14].

Laser treatment of exposed pulp has been shown to improve the outcome of direct pulp capping procedures [15]. Yazdanfar et al. reported a 100% one-year success rate when using diode low-level laser therapy (808 nm, 5 W, continuous wave for 2 s per 1 mm) in direct pulp capping compared to 60% success in non-laser treated group [16].

Similar results were obtained using different laser types in other studies [17, 18]. LLLT application on the pulp–dentine interface after conservative cavity preparation without pulp exposure leads to more aggregated organized collagen fibrils and odontoblast process [19]. Also, the use of carbon dioxide laser on dental pulp of rats induces mineralization [20].

Photobiostimulation by low-level laser application is one of the adjunct aids that are useful to accelerate wound healing through shortening of the inflammatory phase after trauma and optimizing the healing process. LLLT is a noninvasive technique that stimulates biological cell processes. It increases collagen production and mitotic activity of epithelial cells and fibroblasts [21, 22]. LLLT is more efficient in modulating inflammatory mediators including IL-1β and IL-6 and inflammatory cells like neutrophils and macrophages, which are correlated with reduction of the inflammatory process [13, 23, 24], enhanced angiogenesis, and dentinogenesis [25], and expression of growth factors [26].

No study has ever evaluated the pulpal and periapical effect of low-level diode laser application combined with EndoSequence Root Repair material in direct pulp capping procedures. Therefore, the aim of this current study is to evaluate the pulpal and periapical reaction and healing after capping with EndoSequence Root Repair material combined with the application of diode low-level laser in rabbits, using histomorphological analysis.

## Methods

### Sample size

Full effort was done to utilize the least number of animals based on the data collected from the pilot test. The power of the test was calculated using independent t-test. The power of 0.95 was reached for the given value from it with alpha level of 0.05 and minimal sample size of 6 teeth per group for each outcome.

### Animal selection

The study followed the standard practices for biological evaluation of dental materials. All the experiment’s steps followed the European Communities Council Directives of 24th November 1986 (86/906/EEC) recommendations. The study was approved by a Research Ethics Committee, under (Proposal No. 127-09-09). Eight Adult male New Zealand rabbits were selected from the animal housing with age of 8 ± 2 weeks with weight of 2382.5 ± 324.7 g. The animals were kept in polypropylene cages (60 × 45 × 45 cm) in singles under a temperature of 23 ± 2 °C and relative humidity of 55% and 12 h. photoperiod. The experimental procedures were approved by the ethical committee before starting the project with ethical approval no (127-09-19). Laboratory experiments and animal care strictly followed the ethical guidelines of the declaration of Helsinki of the World Medical Association regarding using animals for laboratory experiments.

### Animal grouping

Six out of eight rabbits were selected and randomly distributed into two groups, three rabbits were sacrificed after 2 weeks and considered as group 1 and the other three rabbits were sacrificed after 2 months and considered as group two. Four anterior teeth were included in each animal in each group interval (twelve teeth per each group per interval). Low-level laser was applied to the two lower anterior teeth in all tested animals. The other two animals were sacrificed without intervention to serve as a negative control of pulp tissue baseline reading to compare the changes in tested groups.

### Pulp capping

Animal fasting was done 2 h before surgery. Intra-muscular Pentobarbital injection in dose of 0.5 mg/kg followed by Ketamine hydrochloride injection at a dose of 5 mg/kg (Ketamine10%, Alfasan, Netherland) were administered [27].

The teeth surface was disinfected with 2% chlorhexidine solution (Chlor-X, Prevest DenPro, Jammu, India), using a small cotton pellet. Then teeth were isolated by rubber dam and a class V cavity with a diameter of 1–2 mm was done in the labial surface of anterior teeth with a sterile TC round bur (edednta, Liechtenstein, Switzerland) in 45° to the long axis at low speed of 20,000 rpm and under saline spray coolant. One bur was used for each cavity. Mechanical pulp exposure was performed using endodontic explorer with diameter of 0.15 mm (DG16; Hu-Friedy Co., Chicago, IL). The bleeding was controlled by gentle pressure with sterile cotton pellets [28]. A team of two operators was responsible for all surgical procedures with the aid of 3.5× magnifying dental loupes (Zumax Medical Co Ltd, Suzhou New District, China).

### Low-level laser irradiation

Diode laser of 980 nm wavelength and output power of 0.25 W (Doctor Smile, Lambada Spa, Brendola, Italy) was applied in direct contact technique with the exposure site in continuous mode to the lower teeth for 90 s with a total dose of 1.25 J/cm^2^.

### Pulp capping

Pulp capping was done with regular-set ERRM (ERRM; Brasseler USA, Savannah, GA) using hand pluggers No.2 (Dentsply, Maillefer). After that, cavities were sealed by Glass ionomer (Fuji IXGP; America Inc., Alaip, USA).

### Follow up

The animals were kept on soft diet. The surgical site was examined daily to check the sealing of the filling, abscess formation, or presence of infection. In the meantime, animal health monitoring was performed by observing their normal diet consumption, sleeping hours, and weight [27].

### Histological processing

Animals were sacrificed after 2 weeks and after two months by ketamine overdose. The anterior teeth and the surrounding bone were dissected in buccolingual direction then fixed in 10% buffered formalin with pH of 7.1 for 48 h then samples were subjected to decalcification using Shandon TBD-1 (HCl based; Thermo Electron Corporation, Basingstoke, Hampshire, UK) for 4 weeks at room temperature of 25^0^C. Demineralized specimens were washed under running tap water for 1 h. Histological processing was done after insertion of samples in enclosed perforated cassettes using Excelsior™ AS Tissue Processor (Thermo Fisher Scientific, Fair Lawn, UK). Then paraffin blocks were cut in buccolingual serial sections of 3–4 μm thickness using The Accu-Cut® SRM™ 200 Rotary Microtome (Sakura Finetek, Tokyo, Japan) and sections were stained with hematoxylin and eosin stain (H&E) and Masson Trichrome using Tissue-Tek Prisma device (Sakura Finetek, Tokyo, Japan).

### Histological analysis

Histological analysis was done blindly by two evaluators under light microscope (Eclipse E1000; Nikon, Tokyo, Japan). Images were captured using Nikon DS-Fi2 digital camera at different magnification powers. H&E slides were scanned using IScan Goreo (Ventana Medical System, Roche, Tucson, AZ) [29]. Histological assessment was done for, inflammatory response, odontoblasts cell status, cell density, predentin thickness analysis, presence of vacuoles, and condition of the apical part and periapical area. [28, 30] as follows. The inflammatory response was given grades 1–4, in which grade 1 refers to no or sparse inflammatory cells, grade 2 refers to mild or less than 10 inflammatory cells, grade 3 refers to severe inflammation with abscess or dense infiltration of inflammatory cells that includes more than one third of the pulp space, and grade 4 refers to necrotic pulp [31, 32]. Odontoblast cells status was also given grades 1–4, in which grade 1 refers to palisade odontoblasts, grade 2 refers to the presence of odontoblast cells, and odontoblast-like cells, grade 3 refers to the presence of odontoblast-like cells only, and grade 4 refers to no cells [31, 33]. Regarding predentin thickness analysis, the captured images of predentin at 10× magnification for each group were used for measurements. Predentin thickness was measured in pixels by using ImageJ v1.53e software [31, 34]. All values were calculated from three sites of predentin for each tooth in all groups and we calculated the means and standard deviation. Cell density was evaluated by checking the cells (fibroblasts and undifferentiated mesenchymal cells) density at the central zone of the pulp [35]. The cell counting was performed and analyzed by ImageJ v1.53e software under 40× magnification power.

#### Statistical analysis

The mean and standard deviation for the different groups were calculated first. Welch *t*-test for group-wise comparisons was used. *P* = 0.05 or less was considered for statistical significance. Data were analyzed using SPSS software version 20.0 (IBM Corp., Armonk, NY) and the difference of 0.05 was considered statistically significant.

## Results

### Pulpal histological findings

**Fig. 1:**
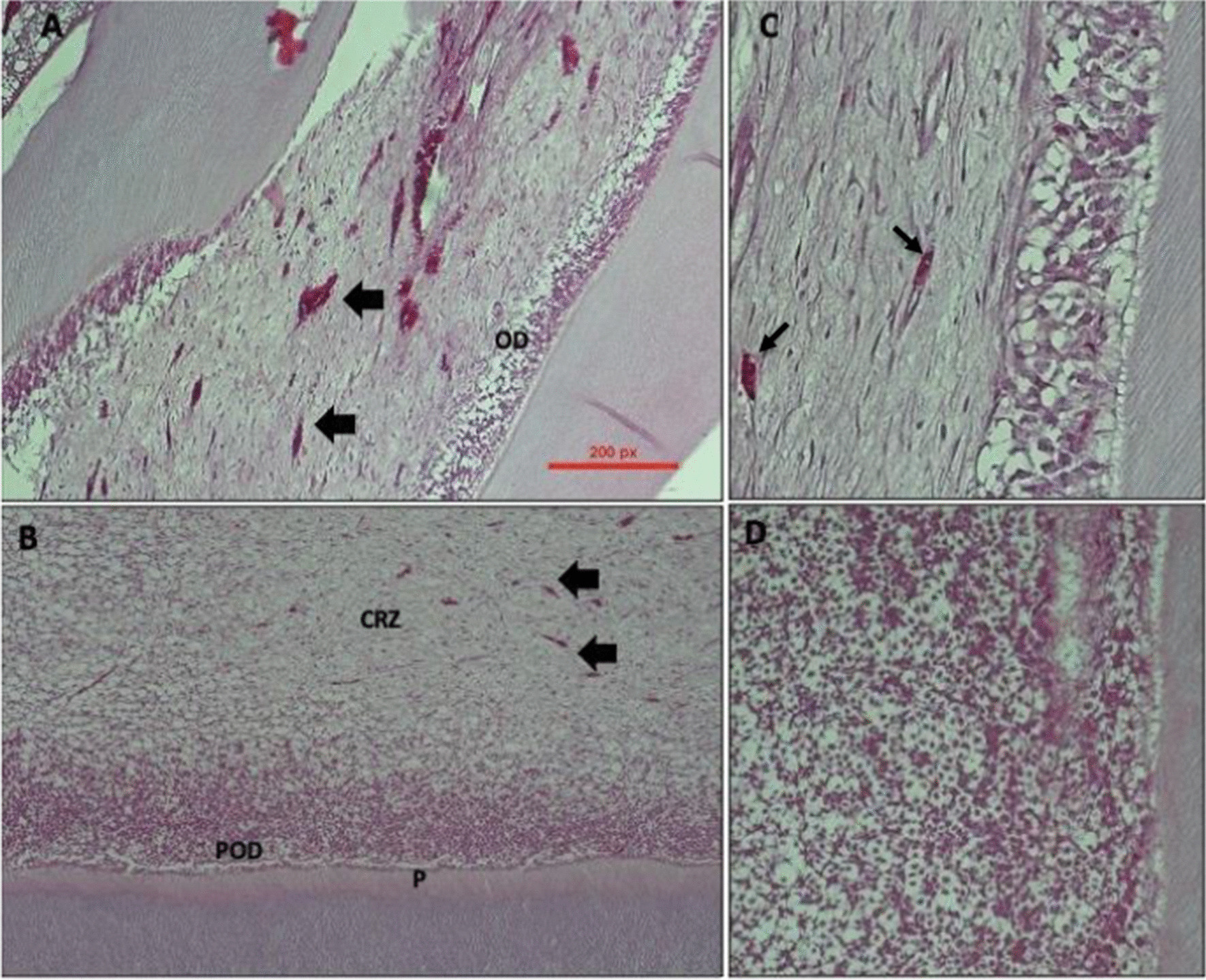
**A**, **C** Images show pulp tissue response after 2 weeks of pulp capping using regular-set ERRM with laser therapy. **A** shows mild inflammation and disorganized odontoblasts (OD) and pre-odontoblastic cells. **C** shows numerous congested Vessels with different sizes. **B**, **D** images show pulp response after 2 weeks of pulp capping using regular-set ERRM with no Laser therapy. **B** the view showed predentin and disorganized pre-odontoblasts cells (POD) with sever inflammation in cell rich zone area (CRZ). The pulp rich cells area showed scattered small size blood vessels (arrows). **D** shows pre odontoblasts (POD) under higher magnification. **A**, **B** are stained with H&E stain and with ×10 magnification. **C**, **D** are stained with H&E with ×20 magnification

After 2 weeks of pulp capping with ERRM material the microscopic images showed mild inflammation and organized palisaded odontoblasts mixed with odontoblast-like cells in teeth treated with laser. But in non-lased teeth, there was severe inflammation detected in the pulp and the disorganized pre-odontoblasts cells found next to dentine wall. The pulp central space of lased teeth exhibited low cellularity and more dilated congested blood vessels while the non-lased teeth showed high cellularity and newly formed small size blood vessels (Fig. 1). The predentin thickness was thicker in the lased group compared to non-lased teeth with a statistically significant difference (*p* < 0.05) (Table 1). Vacuoles were observed near the pulp horn in the coronal part only and were associated with missing odontoblasts. Vacuoles were observed in the control and tested groups. No areas of necrosis or micro-abscess were observed.

**Table 1:** The histomorphometry pulpal features of group one with and without laser therapy

Pulp features	Laser therapy	N	Mean	SD	SE	*p*
Inflammatory response	No	6	2.833	0.408	0.167	0.104
	Yes	4	2.250	0.500	0.250	
Odontoblast cells layer	No	6	2.667	0.516	0.211	0.245
	Yes	4	2.250	0.500	0.250	
Predentin thickness	No	6	11.625	9.907	4.044	0.017*
	Yes	4	25.667	3.748	1.874	
Cell density	No	6	115.000	20.794	8.489	0.368
	Yes	4	158.250	81.246	40.623	

*The mean difference is significant at *p*-value < 0.05

Table 1 shows histological assessment for inflammatory response; odontoblasts cell status; cell density; predentin thickness analysis for group 1 In which the animals were sacrificed after 2 weeks of pulp capping procedures. The predentin thickness was statistically significant (*p* < 0.05) thicker in lased group compared to non-lased teeth. There was no statistically significant difference in the other variables between lased and non-lased groups.

**Fig. 2:**
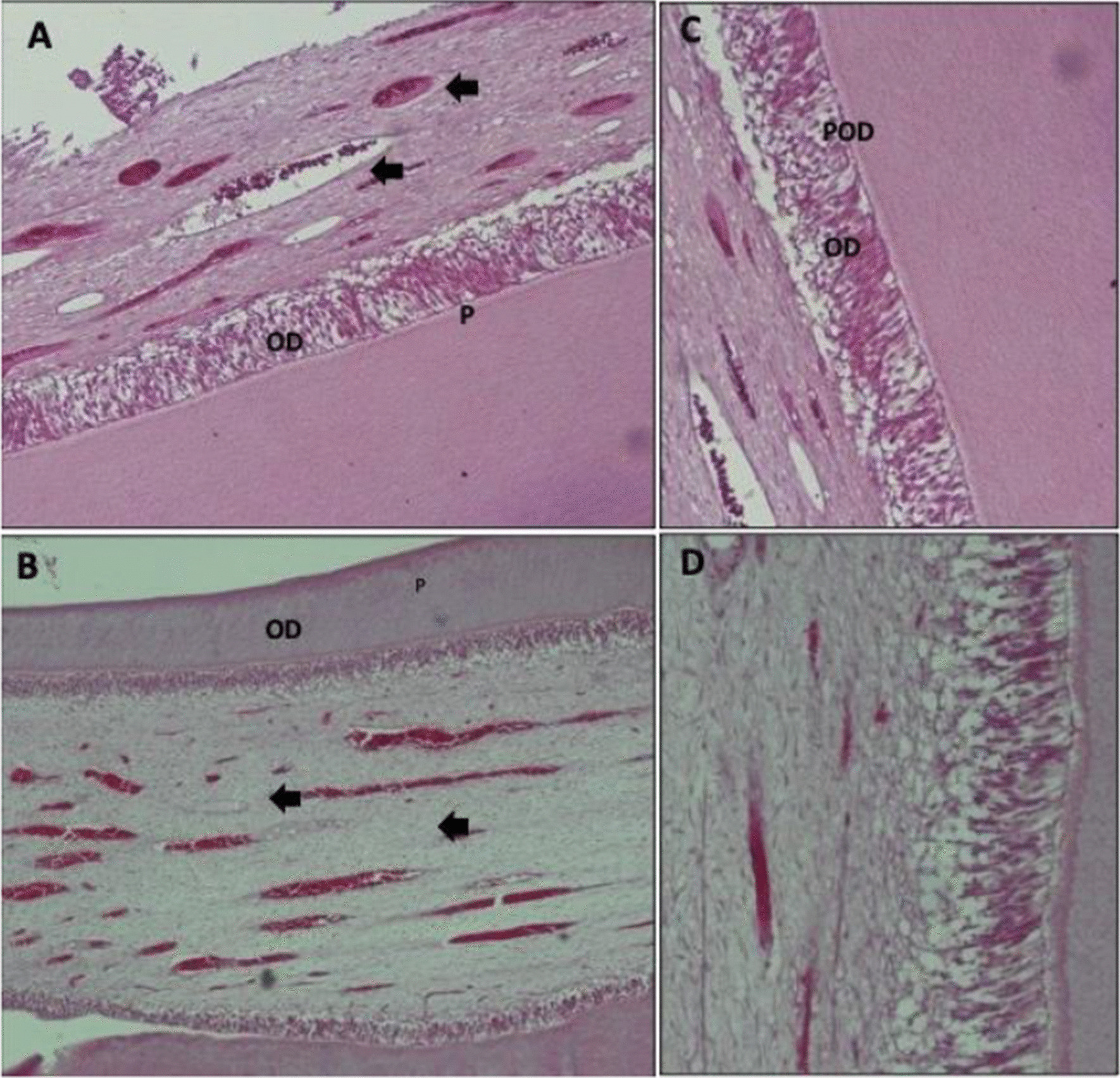
**A**, **C** Show pulp tissue after 2 months of pulp capping using regular-set ERRM with laser therapy. The images of the pulp showed more fibrous tissue with congested numerous dilated blood vessels. Predentin layer is clear and odontoblast with pre-odontoblast cells (OD and POD) are organized and palisaded. **B**, **D** show pulp tissue after 2 months of pulp capping using regular-set ERRM without laser therapy. Images of the pulp showed scares inflammatory cells with congested numerous dilated blood vessels. Predentin layer is clear and odontoblast with pre-odontoblast cells (Insert) are organized and palisaded and. **A, B** are stained with H&E stain and with 10X magnification. **C**, **D** are stained with H&E with 20X magnification

After two months, the inflammatory cells were sparse in both lased and non-lased teeth and the odontoblasts are organized and palisaded. Odontoblast-like cells were more noticeable in the non-lased group. The central pulp zone of lased teeth showed more collagenous fibrous tissue components compared to non-lased teeth but both groups exhibited multiple dilated congested blood vessels (Fig. 2). In Addition, the predentin was observed in the labial side of the teeth. The thickness was variable in the pulpal side, and it was thicker in lased too but no statistically significant difference was found (Table 2).

**Table 2:** The histomorphometry pulpal features of group two with and without laser therapy

Pulp features	Laser therapy	N	Mean	SD	SE	*p*
Inflammatory response	No	7	1.857	0.690	0.261	0.865
	Yes	5	1.800	0.447	0.200	
Odontoblast cells layer	No	7	1.857	0.690	0.261	0.074
	Yes	5	1.200	0.447	0.200	
Predentin thickness	No	7	13.540	6.573	2.484	0.142
	Yes	5	20.043	7.056	3.156	
Cell density	No	7	111.429	58.163	21.984	0.429
	Yes	5	91.200	23.274	10.409	

Table 2 shows histological assessment for inflammatory response; odontoblasts cell status; cell density; predentin thickness analysis for group 2 in which the animals were sacrificed after two months of pulp capping procedures. There was no statistically significant difference in the other variables between lased and non-lased groups.

### Periapical histological findings

**Fig. 3:**
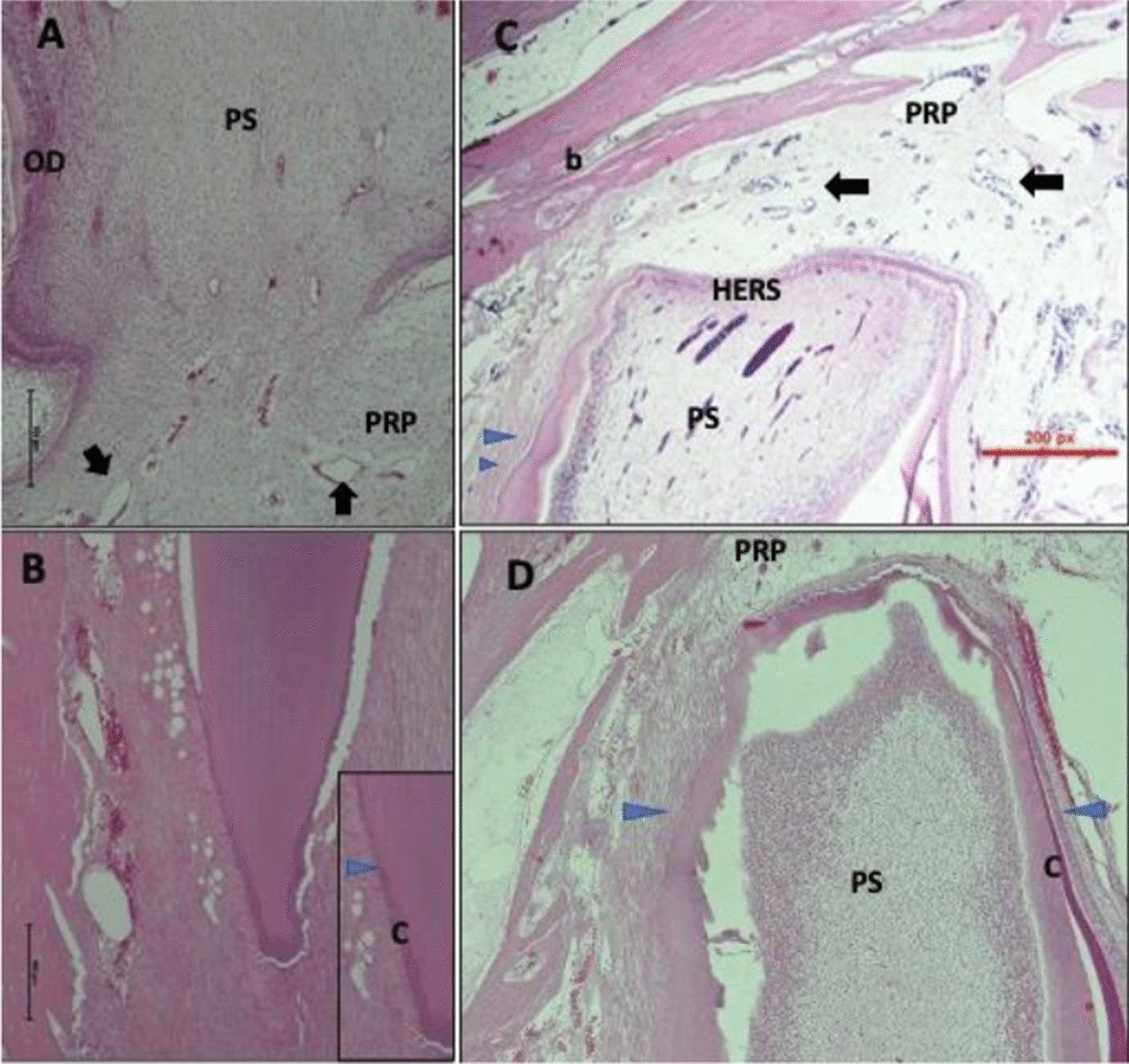
Histological image of periapical area after 2 weeks of pulp capping with (**A** , **B**) or without (**C** , **D**) laser therapy. **A** Incomplete closure of apex of root and the periapical area (PRP) showed fibroblasts with dilated blood vessels (arrows) and mild inflammation. The image also shows odontoblasts (OD) and pulp space (PS) **B**. At complete closure tooth, the periapical area showed thick fibrous tissue with no inflammation and sparse blood vessels. Cementum (c) formed and mature periodontal ligament attached to it (PDL) (blue triangle). **C** in periapical area of incomplete closure of apex, fibroblasts with numerous dilated blood vessels (arrows) with mild inflammation and the Hartwig’s epithelial root sheath (HERS) separate the periapical tissue (PRP) from pulpal tissue. More collagen in apex with immature periodontal ligament (PDL) next to the area of complete dentine formation (blue triangles). No inflammation in the surrounding bone (b) and bone marrow. **D** At complete closure tooth, the periapical area showed fibrosis with numerous blood vessels with no inflammation. Cementum (C) formed and mature PDL (blue triangles) attached in one side and immature PDL in the other side

The histological morphology of the periapical area in group one (2 weeks) with or without laser therapy showed dilated blood vessels either in complete or incomplete apex closure. Although the blood vessels were numerous in periapical tissue, there is no significant infiltration of lymphocytes in the area. In the complete closure, the preapical area shows thick fibrous connective tissue (Fig. 3B, D). In teeth with complete apex closure, the cementum is formed and attached to the mature and well-organized periodontal ligament (Fig. 3B, D), while open apex teeth exhibited irregular PDL attachment (Fig. 3A, C).

**Fig. 4:**
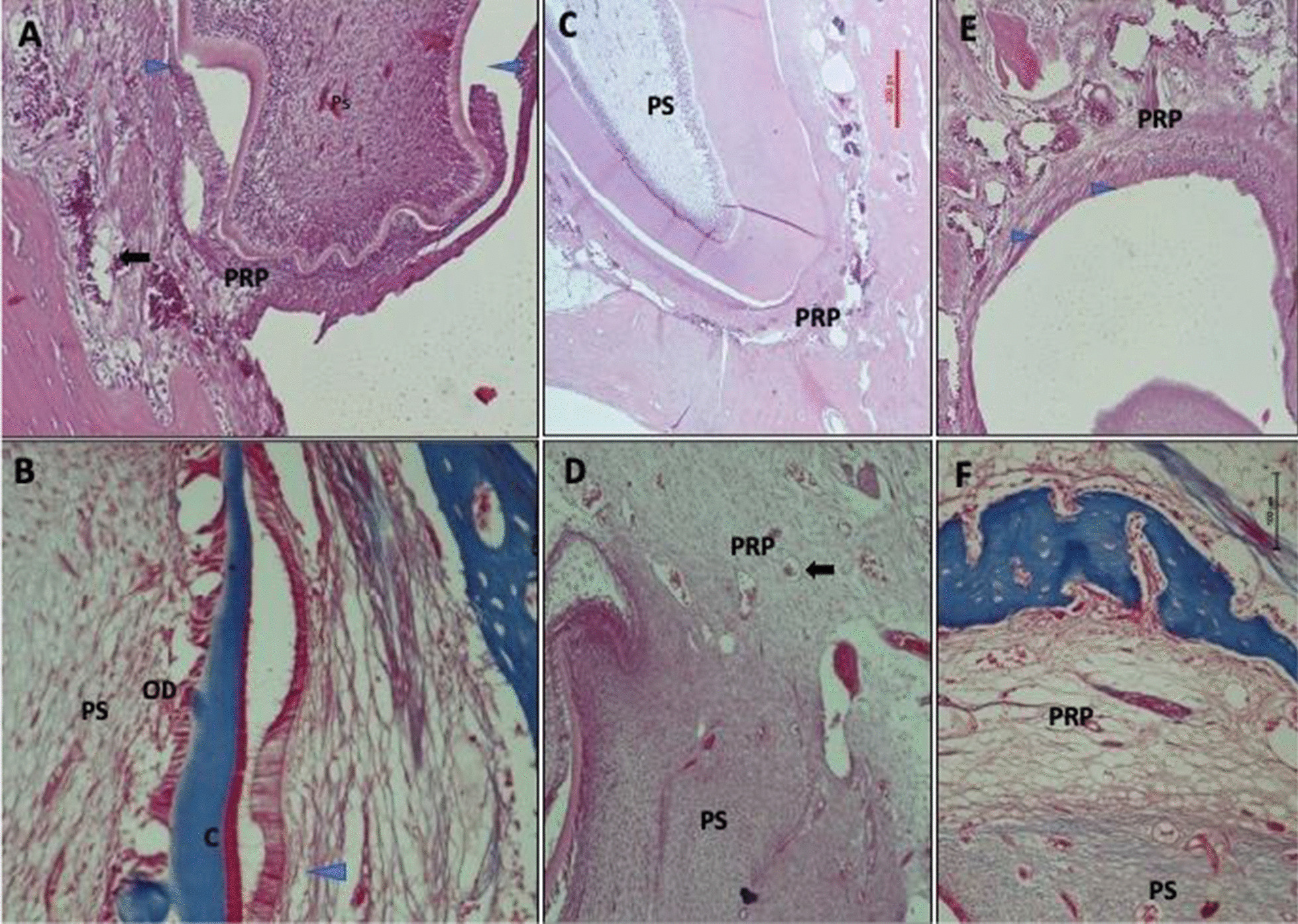
Periapical area after two months of pulp capping with [19] and without (**D**–**F**) laser therapy. **A** Apex continue closing after pulp capping and laser therapy. The periapical area (PRP), fibroblasts with numerous dilated blood vessels (arrow) and mild inflammation. More collagen in periapical and mature PDL next to the area of complete dentine formation and cement formation. Mild inflammation in surrounding bone and bone marrow. **B** Masson Trichome Image showed the mature PDL and cementum. Odontoblasts palisaded in pulpal side (PS) of dentine. **C** with complete closure and thick dentine wall the periapical area showed denser collagen fibrous and normal compact viable bone and blood supply. **D** In teeth without laser therapy, the open apex tooth with neoformed pulpal tissues in the pulp space (PS) do not communicate with the periapical tissues. The clear separation between pulp and periapical tissue is illustrated in Masson trichome stained tissue (**E**). A closed apex on non-lazed teeth showed mature PDL and the periapical area had scattered inflammatory cells with congested blood vessels. **F** Mild inflammation and scattered blood vessels were noticed in periapical area (**D**)

The same was found in group two (Fig. 4), with more numerous maturations of PDL fibers observed with complete development of root apex of the root (Fig. 4A, B). The periapical area of lased teeth showed more fibrosis than non-lased teeth (Fig. 4C, F). Bone in all groups showed normal pattern of growth and viability. In teeth from the non-lased group, there is mild inflammation and scattered blood vessels in the periapical area (Fig. 4D). Closed apex teeth in this group shows mature PDL and scattered inflammatory cells in the periapical area with congested blood vessels (Fig. 4E).

## Discussion

Factors affecting the outcome of pulp capping involve sealing the cavity and the antimicrobial properties of the material that decreases the suspected inflammatory response [4]. All dental materials are irritant at the beginning when they come in direct contact with the tissues and cause inflammatory reactions [36]. Extension of the inflammatory phase after pulp exposure will delay the healing and may compromise successful pulp capping outcome [37]. Therefore, studying pulpal reaction towards the capping material is needed.

The rabbit incisors do not have true anatomical roots. There is a continuation of the growth of rabbit teeth throughout life. Geminal tissue, located at the apices of the teeth, continuously forms enamel to cover each tooth as the tooth constantly grows. This type of teeth is defined as elodont or reserve crowns. They are characterized by absence of differentiation between the crown and root and by having open apices due to continuous growth. The surrounding bone is more cancellous with the presence of adipose tissue that affects healing. The rabbit bone turnover is faster than other rodents. Using rabbits for in vivo experiments has many advantages that include low cost and accepted size [38]. Incisors grow at rate of 2 mm/w. So, a short term of 2 weeks and two months were selected in the current study. Also, rabbit teeth are ideal to examine tissue reaction to material due to their high regenerative power and we can observe the extension of material effect up to the apical part.

The role of low-level lasers in VPT was proven significantly effective. It was reported in a systematic review that VPT combined with lasers exhibited significantly higher success rates than control groups in direct pulp capping procedures [38, 39].

Furthermore, LLLTT was effective in reducing inflammation, promoting cell proliferation [13, 38].

The findings of the present study indicated that predentin thickness increased significantly in the lased group. Therefore, the null hypothesis was rejected. The predentin was most observed in the labial surface due to continuous teeth growth and its thickness varied at the pulpal level which was observed before by Basandi et al. [40]. It was proven that the diode low-level laser produced more aggregated organized collagen fibrils on the pulp–dentine interface [19] and promoted the dentinogenesis process in pulp [41]. In the meantime, it upregulates angiogenic and odontogenic genes [25]. These guarantee the preservation of the dentin structure integrity and performance of the original odontoblasts and the newly formed ones at the exposure site [24, 42]. This explains of the increase of predentin thickness in the lased teeth.

In a randomized clinical trial that evaluated diode laser irradiation combined with a resin-based tricalcium silicate material in direct pulp capping in carious exposures of permanent teeth over a period of 6 months, a statistically significant increase in dentin thickness was found in both groups, laser-treated and nontreated, but clinically diode laser group has shown statistically significant better results [38, 43].

Conversely, in a study that evaluated the effects of a low-level diode laser and dental pulp-capping substances on fibroblasts’ cell proliferation. It was found that when combined with dental pulp-capping materials, LLLTT had no effect on fibroblasts’ cell proliferation [38, 44].

## Conclusion

Using low-level laser after capping of exposed pulp shortens the inflammatory phase and enhances healing. The low-level laser application was capable to preserve dentin integrity. EndoSequence Root Repair Material as a pulp capping material is biocompatible and does not cause a change in the pulp tissues or periapical tissues. The result of this study shows that the combined use of ERRM and low-level laser application can enhance pulp healing and maintain periapical tissues in open and closed apices after pulp capping procedures.

## Data Availability

The datasets used and/or analyzed during the current study are available from the corresponding author on reasonable request. Data cannot be deposited in a public repository since this research is not yet published. For that reason, and for data confidentiality data will be granted upon reasonable request.

## Abbreviations

ERRM: EndoSequence root repair material
LLLT: Low-level laser therapy
OD: Odontoblasts
CRZ: Cell rich zone area
POD: Pre-odontoblasts cells
PRP: Periapical area
PS: Pulp space
c: Cementum
PDL: Periodontal ligament
HERS: Hartwig’s epithelial root sheath
